# Predictive value of CT and ^18^F-FDG PET/CT features on spread through air space in lung adenocarcinoma

**DOI:** 10.1186/s12885-024-12220-x

**Published:** 2024-04-08

**Authors:** Haijun Li, Lifeng Li, Yumeng Liu, Yingke Deng, Yu Zhu, Ling Huang, Ting Long, Li Zeng, Yongqiang Shu, Dechang Peng

**Affiliations:** 1 Department of Radiology, The First Affiliated Hospital, Jiangxi Medical College, Nanchang University, No.17 Yongwai Zheng Street, Donghu District, Nanchang City, 330006 Jiangxi Province China; 2 PET Center, The First Affiliated Hospital, Jiangxi Medical College, Nanchang University, Nanchang, Jiangxi Province China; 3 Department of Radiology, The Affiliated Changsha Central Hospital, Hengyang Medical School, University of South China, Changsha, Hunan Province China

**Keywords:** Lung adenocarcinoma, Computed tomography, ^18^F-FDG PET/CT, STAS, Surgery

## Abstract

**Background:**

Lung adenocarcinoma, a leading cause of cancer-related mortality, demands precise prognostic indicators for effective management. The presence of spread through air space (STAS) indicates adverse tumor behavior. However, comparative differences between ^18^F-fluorodeoxyglucose (^18^F-FDG) positron emission tomography(PET)/computed tomography(CT) and CT in predicting STAS in lung adenocarcinoma remain inadequately explored. This retrospective study analyzes preoperative CT and ^18^F-FDG PET/CT features to predict STAS, aiming to identify key predictive factors and enhance clinical decision-making.

**Methods:**

Between February 2022 and April 2023, 100 patients (108 lesions) who underwent surgery for clinical lung adenocarcinoma were enrolled. All these patients underwent ^18^F-FDG PET/CT, thin-section chest CT scan, and pathological biopsy. Univariate and multivariate logistic regression was used to analyze CT and ^18^F-FDG PET/CT image characteristics. Receiver operating characteristic curve analysis was performed to identify a cut-off value.

**Results:**

Sixty lesions were positive for STAS, and 48 lesions were negative for STAS. The STAS-positive was frequently observed in acinar predominant. However, STAS-negative was frequently observed in minimally invasive adenocarcinoma. Univariable analysis results revealed that CT features (including nodule type, maximum tumor diameter, maximum solid component diameter, consolidation tumor ratio, pleural indentation, lobulation, spiculation) and all ^18^F-FDG PET/CT characteristics were statistically significant difference in STAS-positive and STAS-negative lesions. And multivariate logistic regression results showed that the maximum tumor diameter and SUVmax were the independent influencing factors of CT and ^18^F-FDG PET/CT in STAS, respectively. The area under the curve of maximum tumor diameter and SUVmax was 0.68 vs. 0.82. The cut-off value for maximum tumor diameter and SUVmax was 2.35 vs. 5.05 with a sensitivity of 50.0% vs. 68.3% and specificity of 81.2% vs. 87.5%, which showed that SUVmax was superior to the maximum tumor diameter.

**Conclusion:**

The radiological features of SUVmax is the best model for predicting STAS in lung adenocarcinoma. These radiological features could predict STAS with excellent specificity but inferior sensitivity.

## Highlights

• Evaluated PET/CT and CT features for predicting STAS in lung adenocarcinoma.

• SUVmax and maximum tumor diameter were independent influential factors for STAS(+) in lung adenocarcinoma.

• SUVmax showed higher predictive efficacy than maximum tumor diameter.

• Radiological characteristics may serve as noninvasive biomarkers for STAS prediction in lung adenocarcinoma.

## Introduction

Lung cancer is a highly lethal malignancy and is the leading cause of cancer-related deaths worldwide. The Overall five-year lung cancer survival is relatively low compared to other major malignancies. Early diagnosis and proper treatment of lung cancer are crucial for enhancing patient survival [1, 2]. Lung adenocarcinoma, which is the predominant form of lung cancer, is known as the most fatal cancer globally [3]. With the popularization of imaging tests, more and more lung adenocarcinomas are detected at an early stage. The standard surgery for lung adenocarcinoma is usually anatomical lobectomy and segmental resection or wedge resection can be chosen in combination with the patient's metastatic lymph node status. The spread through air space (STAS) was introduced in the 4th edition of the World Health Organization classification of thoracic tumors in 2015, and was considered as another mode of tumor spread in lung adenocarcinoma [4]. It was defined as micropapillary clusters, solid nests, or single cells that extend beyond the tumor margins into the airspace of the surrounding lung parenchyma, and tumor cells can detach from the main tumor, migrate through the air space, and then reattach to the distant alveolar wall through vascular symbiosis [5]. A study revealed that STAS was a poor prognostic predictor and an aggressive pathological characteristic [2]. STAS represented a detrimental prognostic indicator for both limited resection and radical resection of lung cancer, exerting a negative impact on patient outcomes, and is a prognostic risk factor for local recurrence [6, 7]. Therefore, predicting STAS was useful for developing clinical diagnoses, and treatment plans, and facilitating surgical decision-making for patients. A previous study found that using computed tomography(CT) features to predict STAS had a high sensitivity of 89.2% and specificity of 60.3% and identified the percentage of solid components as an independent predictive factor for STAS [8]. Another study utilized ^18^F-fluorodeoxyglucose (^18^F-FDG) positron emission tomography(PET)/CT to predict STAS and found that maximum standardized uptake value (SUVmax) was a valuable indicator for predicting STAS in clinical stage I lung adenocarcinoma [9]. However, there is currently no research comparing the differences between ^18^F-FDG PET/CT and CT in predicting STAS in lung adenocarcinoma. The purpose of this retrospective study was to investigate the usefulness and difference of CT and ^18^F-FDG PET/CT features in predicting and evaluating the STAS status of lung adenocarcinoma, to provide predictive value for imaging in the clinical diagnosis and treatment of lung adenocarcinoma.

## Material and methods

The Ethics Committee of The First Affiliated Hospital of Nanchang University approved this retrospective study, and the requirement for informed consent from participants was not required. The records and information of the patients were anonymized and de-identified before analysis.

### Subjects

In this retrospective analysis, we reviewed patients diagnosed with lung adenocarcinoma, who were hospitalized in the Department of Thoracic Surgery between February 2022 and April 2023. All patients had a lung lesion initially detected by a thin-section chest CT scan and confirmed as lung adenocarcinoma through CT-guided percutaneous needle biopsy or endobronchial ultrasound-guided transbronchial needle aspiration. An enhanced magnetic resonance imaging scan of the brain was used to exclude brain metastatic lesions. Clinical staging was done through ^18^F-FDG PET/CT within 2 weeks before surgery and the choice of surgical modality (lobectomy, segmentectomy, or wedge resection) was made based on the results of CT and ^18^F-FDG PET/CT. The pathological classification criteria of the International Association for the Study of Lung Cancer, the American Thoracic Society, and the European Respiratory Society were used [10]. Using thin-slice CT (slice thickness = 1 mm) scans, ground glass nodules (GGN) were defined as areas of pulmonary haze attenuation with preserved bronchial and vascular margins, and solid components were defined as plaques completely obscuring the lung parenchyma, with some solid nodules having features of both GGN and solid components. Participants meeting one of the following criteria will be excluded from the study cohort: history of prior lung surgery, prior neoadjuvant chemotherapy for malignancy, no thin-slice CT, ^18^F-FDG PET/CT image data or pathology results; receive chemotherapy before CT or ^18^F-FDG PET/CT, significant distant metastases on preoperative images; and beyond pathological T2b or N2.

### Clinical and pathologic characteristics

The medical records and postoperative pathology reports of all patients were reviewed. Clinicopathologic features included age, sex, carcinoembryonic antigen (CEA) before surgery, smoking status, way of operation, pathological overall size, histological subtype (including minimally invasive adenocarcinoma (MIA), lepidic predominant, acinar predominant, papillary predominant, micropapillary predominant and solid predominant) (WHO classification 2015), T stage (T1a/b/c, T2a/b), N stage (N0-2), visceral pleural invasion (VPI), lymphovascular invasion (LVI) and perineural invasion.

The tumors were classified according to the definitions provided by the World Health Organization and staged according to the tumor-node-metastasis classification described in the American Joint Committee on Cancer Staging Manual (8th edition) [11]. Surgically resected specimens were fixed in 10% formalin, placed on paraffin blocks, sectioned 4 μm thick, and stained with hematoxylin and eosin. STAS was defined as pathological micropapillary clusters separated from the main tumor, solid nests separated from the main tumor, or single cells beyond the edge of the tumor. All tissue sections were interpreted by a pulmonary pathologist with more than 10 years of experience. All identified lesions were classified into STAS-positive/negative categories based on pathological findings.

### CT Acquisition and interpretation

The patient was positioned supine for the CT scan, which was performed at the end of the inspiration phase. The thin-section CT scan was performed using one of the following CT scanners: SOMATOM Force (Siemens Healthcare) or Brilliance iCT (Philips Healthcare) were utilized. This study utilized the following protocol: Specifically, Siemens Healthcare utilized 130 kV, with a range of 30–150 mAs and 5 mm slice thickness, while Philips Healthcare utilized 120 kV, with a range of 60–120 mAs and 5 mm slice thickness. The reconstructed slice thickness was 1 mm. Two experienced radiologists analyzed the CT images independently and reached a consensus through discussion when disagreements arose. Neither radiologist was aware of the presence or absence of STAS.

All data were measured three times and averaged on a cross-sectional image of the chest CT with pictures archived in a communication system (PACS) (lung window setting: width 1,500 HU, horizontal -600 HU; mediastinal window setting: width 350 HU, horizontal 40 HU). We then evaluated the maximum tumor diameter, maximum solid component diameter, consolidation/tumor ratio (CTR), tumor location(central or peripheral), type of nodules (solid, part-solid, or GGN), margin(smooth or blurred), shape (round to oval or irregular), vascular convergence, air bronchogram, pleura indentation, cavitation, lobulation, spiculation, and ill-defined peripheral opacity. The CTR was defined as the rate of the maximum consolidation (C) diameter divided by the maximum tumor (T) diameter of the lung window. All patients enrolled underwent an HRCT scan within 2 weeks before surgery.

### ^18^F-FDG PET/CT

^18^F-FDG PET/CT (Discovery MI, GE Healthcare, Waukesha, WI, USA) was performed within 2 weeks before surgical resection, and all patients were fasted for at least 6 h and injected intravenously with ^18^F-FDG (3.7–5.5 MBq/kg) before resting for approximately 60 min for PET/CT examination. PET/CT imaging was performed 60 min after radiotracer injection. Initially, CT images were obtained without contrast enhancement, ranging from the cranial base to the upper thigh, using parameters of 120 kVp and a 3.75 mm slice thickness. PET/CT images were obtained in a three-dimensional list mode, with 5–6 bed positions and a duration of 1.5 min per bed. The acquired PET/CT images were reconstructed on 128 × 128 matrices using an ordered subset expectation maximization algorithm with 2 iterations and 8 subsets. CT-based attenuation correction was also utilized during the reconstruction process.

The evaluation of ^18^F-FDG PET/CT was performed using the PETVCAR (PET volume computed assisted reading, GE Healthcare) software which was run on the AW4.7 post-processing workstation. ^18^F-FDG PET/CT metabolic parameters included standardized uptake value (SUV, SUVmax, SUVmean, and SUVpeak), standard uptake value of lean body mass (SUL, SULmax, SULmean, SULpeak), metabolic tumor volume (MTV), and total lesion glycolysis (TLG). SUVmax, SUVmean, and SUVpeak represent the maximum, mean, or peak values of the volume of interest, respectively. SULmax, SULmean, and SULpeak represent the maximum, mean, or peak standardized uptake values normalized to lean body mass, respectively. MTV, defined as the FDG receptor tumor volume, was automatically measured by a volumetric computer-assisted reading program with a threshold of SUVmax 40% [12]. TLG was calculated using the formula: [MTV × SUVmean] [13].

### Statistical analysis

Statistical analyses were conducted using IBM SPSS Statistics Software (IBM version 21). Quantitative data were presented as mean ± standard deviation. Count data was represented using composition ratios. Comparisons of clinical, pathologic, and CT or ^18^F-FDG PET/CT features between the STAS-positive and STAS-negative groups were performed using the χ^2^ test or Fisher exact test for categorical variables and the Student *t* test or Mann–Whitney U test for continuous variables. The  *p*-value <0.05 was considered statistically significant.

Univariate and multivariate regression analyses of characteristic differences between CT and PET/CT were carried out to obtain predictive indicators of STAS, which assisted in preoperative diagnosis and treatment decision-making. Variables with* p *<0.05 in univariate analyses were included in multivariate analyses. The classifier was used to obtain the average receiver operating characteristic (ROC) and the average area under the curve (AUC). The AUC was a metric used to compare the predictive performance of different models. The ROC curve was a graphical representation used to evaluate the performance of a binary classifier at varying threshold settings and to determine the cutoff value. To determine the best cutoff value, the Youden index, which was the difference between the sensitivity and the false positive rate, is maximized. Moreover, the DeLong test was conducted to examine the variations in AUC between the ROC curves of SUVmax and maximum tumor diameter, aiming to evaluate and compare their discriminative capacities in predicting STAS. A *p*-value < 0.05 was deemed indicative of statistically significant differences, highlighting substantial distinctions in predictive performance between SUVmax and maximum tumor diameter.

## Results

### Clinical characteristics

**Fig. 1:**
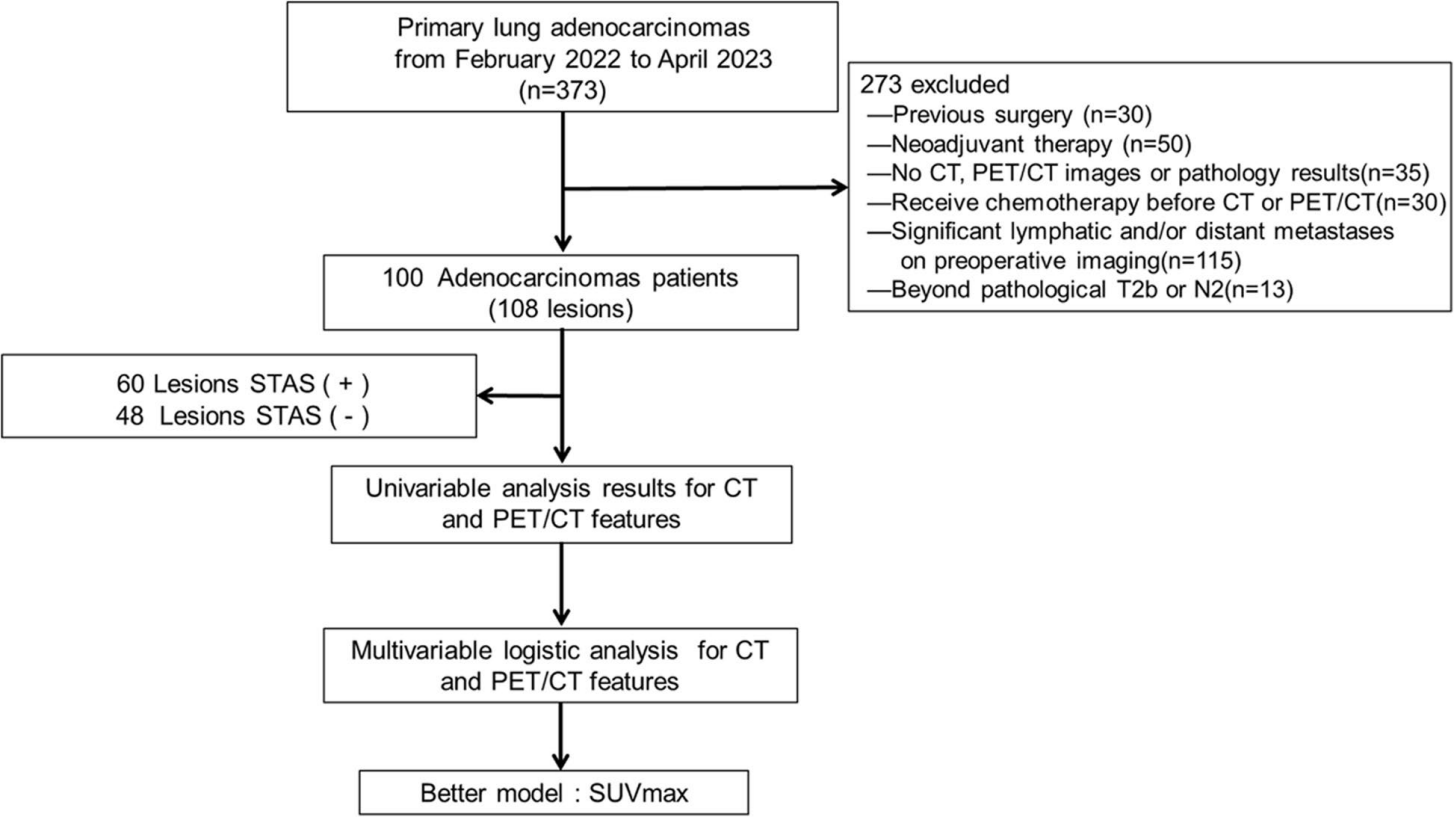
The flow diagram showed the patient selection and exclusion criteria. Note: STAS, spread through air space, STAS ( +), STAS-positive; STAS (-), STAS-negative; SUVmax, maximum standardized uptake value

Three hundred seventy-three patients diagnosed with lung adenocarcinoma were screened in the study. With 30 previous surgery, 50 cases due to prior neoadjuvant therapy, 35 lack of CT, ^18^F-FDG PET/CT or pathology data, 30 cases receiving chemotherapy before CT or ^18^F-FDG PET/CT, 115 significant lymphatic and/or distant metastases on preoperative imaging, and 13 beyond pathological T2b or N2 were excluded. After the screening, only 100 patients (108 lesions), both STAS-positive or negative lesions, who had undergone pathologically confirmed lung adenocarcinoma were finally included in the study (Fig. 1).

The clinical characteristics of the subjects were presented in Table 1. The study population consisted of 48 males and 52 females with a total of 108 lesions (including 60 STAS-positive and 48 STAS-negative and a mean age of 62.7 years. Lobectomy was performed for 72 lesions, segmentectomy for 25 lesions, and wedge resection for 11 lesions. There were no significant differences observed in smoking and CEA < 5 levels between the two groups. A higher proportion of STAS-positive patients (75%) underwent lobectomy (75.0%, 45/60 lesions) compared to STAS-negative (56.3%, 27/48 lesions). Additionally, all patients had negative surgical margins.

**Table 1:** Clinical characteristics of our study patients

**Characteristic**	**Patients**	**STAS(+)**	**STAS(-)**	**t/χ**^**2**^** value**	***p-*value**
**Age (years, *n *= 100)**	62.7±10.4	64.1±9.4	60.9±11.5	1.524	0.131
**Sex (*n*=100)**				8.431	**0.004**
Male	48	35	13		
Female	52	23	29		
**CEA (ng/mL)**				0.029	0.865
<5	85	49	36		
≥5	15	9	6		
**Clinical stage**				11.101	**0.004**
I	84	41	43		
II	16	15	1		
IIIA	8	4	4		
**Smoking status (*n *= 100**)				0.004	0.948
Never	55	30	22		
Former or current	45	28	20		
**Operation (*n *= 108)**				10.196^a^	**0.006**
Lobectomy	72	45	27		
Segmentectomy	25	7	18		
Wedge resection	11	8	3		

*Note*: ^a^Fisher exact test; *STAS *spread through air space, *STAS (+) *STAS-positive, *STAS (-) *STAS-negative, *CEA *carcinoembryonic antigen. Values are presented as mean ± standard deviation unless otherwise indicated. *p* value < 0.05 indicates a statistically significant difference

### Pathologic features

The pathological features of STAS-positive (*n* = 60) and STAS-negative (*n* = 48) were summarized in Table 2. A statistically significant relationship was found between STAS and histological subtypes of lung adenocarcinoma. The most common type of STAS positive was acinar predominant (41 of 60, 68.3%), while STAS negative cases were characterized by MIA (16/48, 33.3%). Apart from the histological subtype, STAS status also showed differences in pathological overall size (*p* < 0.001), pathological T stage (*p* = 0.008), and pathological N stage (*p* = 0.002). In addition, there were significant statistical differences in visceral pleural invasion and lymphovascular invasion, but not in perineural invasion.

**Table 2:** Pathologic characteristics

**Characteristic**	**All patients**	**STAS (+)**	**STAS (-)**	**t/χ**^2^** value**	***p*-value**
**Pathological overall size**	2.2 ± 1.0	2.5 ± 1.0	1.8 ± 0.8	3.869	** < 0.001**
**Histological subtype**				26.803^a^	** < 0.001**
MIA	16	0	16		
Lepidic predominant	13	8	5		
Acinar predominant	65	41	24		
Papillary predominant	6	4	2		
Micropapillary predominant	2	1	1		
Solid predominant	6	6	0		
**T stage**				13.365^a^	**0.008**
T1a	10	1	9		
T1b	41	22	19		
T1c	37	21	16		
T2a	14	11	3		
T2b	6	5	1		
**N stage**				10.800^a^	**0.005**
N0	88	44	44		
N1	12	12	0		
N2	8	4	4		
**VPI**				8.727	**0.003**
Present	91	45	46		
Absent	17	15	2		
**LVI**				9.060	**0.003**
Present	29	23	6		
Absent	79	37	42		
**Perineural invasion**				0.042^a^	0.838
Present	6	3	3		
Absent	102	57	45		

*Note*: ^a^Fisher exact test; *STAS* Spread through air space, *STAS (* +*)*STAS-positive, *STAS (-)*STAS-negative, *MIA* minimally invasive adenocarcinoma, *VPI* visceral pleural invasion, *LVI* lymphovascular invasion. The *p* value < 0.05 indicates a statistically significant difference

### CT Features and ^18^F-FDG PET/CT by STAS status

**Fig. 2:**
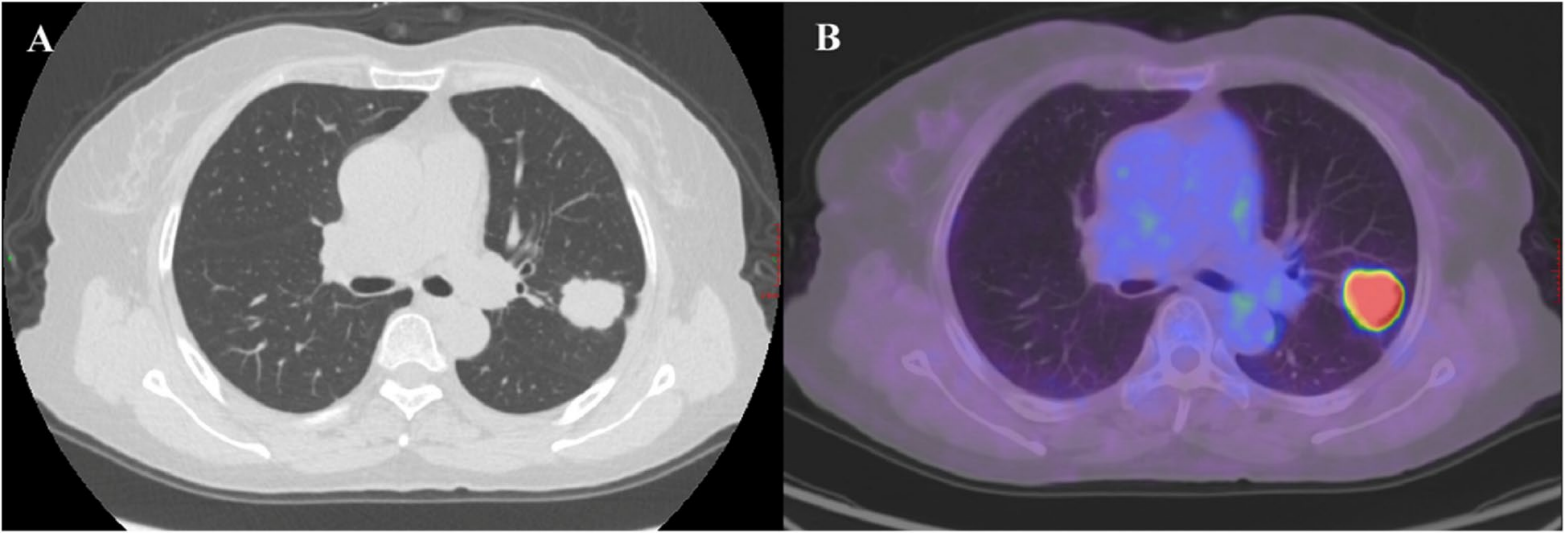
A typical CT and ^18^F-FDG PET/CT images of STAS-positive lung adenocarcinoma in a 55-year-old female patient. The histological subtype was of a lower grade (solid predominant). **A** The CT scan showed a solid density nodule in the upper lobe of the left lung, with a maximum diameter of approximately 2.9 cm. The nodule appears lobulated and have fine spiculations around it. It was causing traction on the adjacent pleura. There was no calcification present, and no ill-defined peripheral opacity. **B** The PET/CT scan showed abnormal uptake of FDG, with corresponding values of SUVmax, SUVmean, SUVpeak, SULmax, SULmean, SULpeak, MTV and TLG was 11.2, 7.3, 9.1, 8.2, 5.3, 6.6, 7.9 and 57.8, respectively

**Fig. 3:**
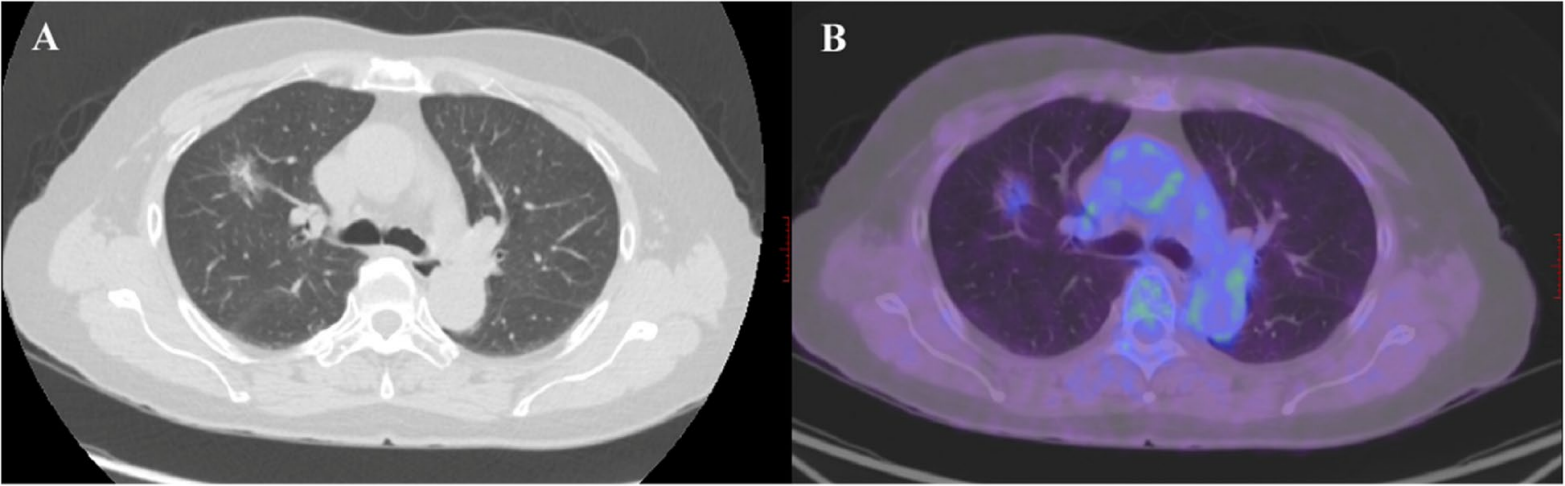
A typical CT and ^18^F-FDG PET/CT images of STAS-negative lung adenocarcinoma in a 62-year-old female patient. The histological subtype was of an intermediate grade (acinar predominant predominant). **A** The CT scan showed a mixed ground-glass density nodule in the upper lobe of the right lung, with a maximum diameter of approximately 2.2 cm. The solid component within the nodule measures 0.6 cm, accounting for approximately 27.3% of the total volume. The nodule have clear borders and no spiculations or traction on the pleura. **B** The PET/CT scan showed slight uptake of FDG, with corresponding values of SUVmax, SUVmean, SUVpeak, SULmax, SULmean, SULpeak, MTV and TLG was 2.4, 1.3, 1.6, 1.8, 1.0, 1.2, 2.6 and 3.4, respectively

The CT and ^18^F-FDG PET/CT features of all subjects with STAS status were shown in Tables 3 and 4, respectively. The typical CT and ^18^F-FDG PET/CT images of lung adenocarcinoma in STAS-positive and STAS-negative patients were shown in Figs. 2 and 3, respectively. In Table 3, the results of the univariate analysis of CT features found pleura indentation, lobulation, and spiculation were more frequent in STAS-positive patients, while there were no differences in other CT features including vascular convergence, air bronchogram, and ill-defined peripheral opacity. Significant differences in the type of nodules were observed. STAS-positive tumors had a larger maximum tumor and maximum solid component diameter than STAS-negative tumors. The CTR levels were higher in STAS-positive tumors. Among the 60 lesions in the STAS-positive group, 42 were solid nodules, 16 were part-solid, and 2 were GGN. The STAS-negative group consisted of 48 lesions, of which 16 were solid nodules, 23 were part-solid nodules, and 9 were GGN. All features of ^18^F-FDG PET/CT features including SUVmax, SUVmean, SUVpeak, SULmax, SULmean, SULpeak, MTV, and TLG demonstrated significant increases in STAS-positive lesions compared to STAS-negative patients (*p* < 0. 05) (Table 4).

**Table 3:** Univariable Analysis Results for CT features

**Characteristic**	**All patients**	**STAS (+)**	**STAS (-)**	**t/χ**^**2**^ value	***p*-value**
**Tumor location**				1.964^a^	0.147
Central	9	7	2		
Peripheral	99	53	46		
**Type of nodules**				16.233	** < 0.001**
Solid	58	42	16		
Part-solid	39	16	23		
GGN	11	2	9		
**Maximum tumor diameter, cm**	2.2 ± 0.9	2.4 ± 0.9	1.9 ± 0.7	3.400	**0.001**
**Maximum solid component diameter, cm**	1.7 ± 1.1	2.2 ± 1.0	1.2 ± 1.0	4.990	** < 0.001**
**CTR**				22.377	** < 0.001**
CTR = 1	58	42	16		
0.5 ≤ CTR < 1.0	28	15	13		
0 ≤ CTR < 0.5	22	3	19		
**Shape**				0.007	0.931
Round to oval	49	27	22		
Irregular	59	33	26		
**Margin**				0.347	0.556
Smooth	71	38	33		
Blurred	37	22	15		
**Vascular convergence**				2.779	0.095
Present	70	43	27		
Absent	38	17	21		
**Air bronchogram**				2.067	0.15
Present	12	9	3		
Absent	96	51	45		
**Pleural indentation**				12.188	** < 0.001**
Present	71	48	23		
Absent	37	12	25		
**Cavitation**				1.463^a^	0.339
Present	97	52	45		
Absent	11	8	3		
**Lobulation**				6.492	**0.011**
Present	88	54	34		
Absent	20	6	14		
**Spiculation**				5.027	**0.025**
Present	75	47	28		
Absent	33	13	20		
**Ill-defined peripheral opacity**				0.697	0.404
Present	43	26	17		
Absent	65	34	31		

*Note*: ^a^Fisher exact test; *STAS* Spread through air space, *STAS (* +*)*STAS-positive, *STAS (-)*STAS-negative, *CTR* consolidation/tumor ratio

**Table 4:** Univariable Analysis Results for ^18^F-FDG PET/CT features

Characteristic	All patients	STAS( +)	STAS(-)	t value	*p*-value
SUVmax	6.0 ± 5.0	8.2 ± 5.1	3.3 ± 3.2	5.686	< 0.001
SUVmean	3.7 ± 3.2	5.1 ± 3.4	2.0 ± 2.0	5.540	< 0.001
SUVpeak	4.2 ± 3.6	5.7 ± 3.7	2.3 ± 2.5	5.381	< 0.001
SULmax	4.7 ± 4.1	6.4 ± 4.3	2.6 ± 2.5	5.449	< 0.001
SULmean	2.9 ± 2.6	4.0 ± 2.8	1.5 ± 1.6	5.449	< 0.001
SULpeak	3.3 ± 3.0	4.6 ± 3.1	1.8 ± 1.9	5.419	< 0.001
MTV, cm^3^	3.6 ± 3.5	4.3 ± 4.2	2.7 ± 2.0	2.573	0.012
TLG	16.2 ± 29.7	23.1 ± 36.0	7.6 ± 15.8	2.764	0.007

*Note*: *STAS* spread through air space, *STAS (* +*)*STAS-positive, *STAS (-)*STAS-negative, *SUV* standardized uptake value, *SUL* standardized uptake value normalized by lean body mass, *MTV* metabolic tumor volume, *TLG* total lesion glycolysis

### Imaging predictors of STAS status in lung adenocarcinoma

Variables with a *p*-value less than 0.05 in the univariable analysis of CT features (model 1) (Table 3) including the type of nodules, maximum tumor diameter, maximum solid component diameter, CTR, presence of pleural indentation, lobulation, spiculation, along with all ^18^F-FDG PET/CT features (model 2) (Table 4) was included in the multivariable logistic regression and the receiver operating characteristic (ROC) curve analyses (Table 5). The CT features: Maximum tumor diameter (odds ratio, 2.656; 95% confidence interval: 1.673, 4.218) remained an independent predictor of STAS in multivariable analyses in model 1, whereas the ^18^F-FDG PET/CT features SUVmax (odds ratio, 1.348; 95% confidence interval: 1.180, 1.541) was independent predictors of STAS in model 2.

**Table 5:** Multivariable logistic analysis results for CT and ^18^F-FDG PET /CT features

Characteristics	Odds Ratio(95% CI)	*p-*value
**Model 1: CT features**		
Maximum tumor diameter	2.656 (1.673, 4.218)	< 0.001
**Model 2: **^**18**^**F-FDG PET/CT features**		
SUVmax	1.348 (1.180, 1.541)	< 0.001

*Note*: *SUVmax* Maximum standardized uptake value, *95%CI* 95% confidence interval

### ROC curve analysis

**Fig. 4:**
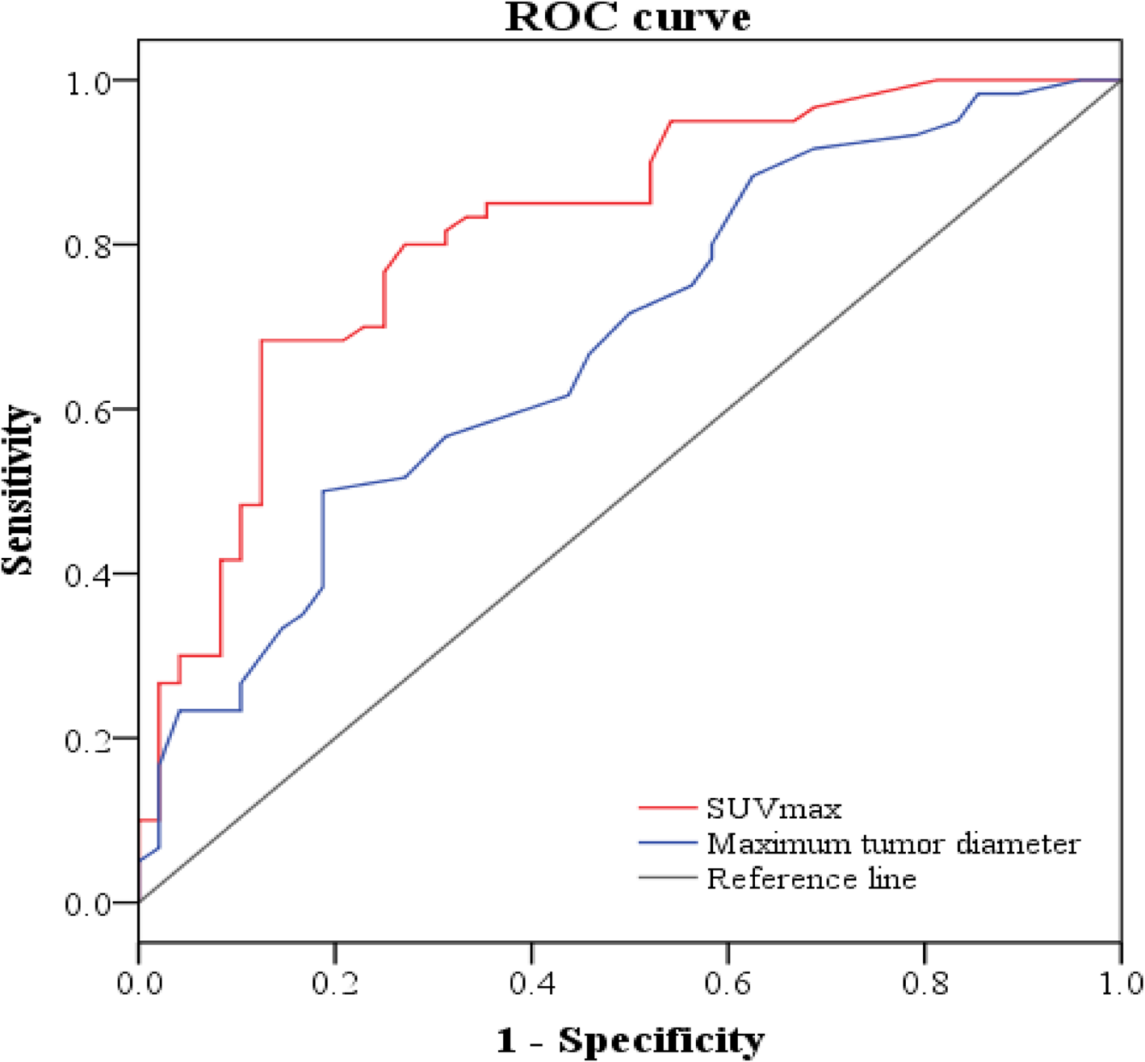
Receiver operating characteristic (ROC) curves showed that the mean area under the curve (AUC) was greater in the model that used the SUVmax of ^18^F-FDG PET/CT than in the model that used the maximum tumor diameter for predicting spread through air spaces (0.82 vs. 0.68). Note: ROC, receiver operating characteristic; SUV, standardized uptake value

Among all lesions, the ROC curve was plotted for SUVmax of PET/CT and the maximum tumor diameter of CT (Fig. 4), and the area under the curve (AUC) was 0.82 (95% CI: 0.74–0.90) for SUVmax vs. 0.68 (95% CI: 0.58–0.78) for maximum tumor diameter respectively. The cut-off value for SUVmax was 5.05 with a sensitivity of 68.3% and a specificity of 87.5%. The cut-off value for maximum tumor diameter was 2.35 with a sensitivity of 50.0% and a specificity of 81.2%. The ROC curves of the two groups underwent a DeLong test, yielding a statistically significant difference with *z* = 2.699 and *p* = 0.0069. This statistical outcome indicates a discernible distinction between the two sets of curves. Notably, the prediction efficacy of STAS based on PET/CT SUVmax surpassed that of the maximum tumor diameter of CT features.

## Discussion

Previous studies have mainly focused on using CT or ^18^F-FDG PET/CT alone to predict the presence of STAS. To the best of our knowledge, this is the first study to use a combination of ^18^F-FDG PET/CT and CT imaging features to predict STAS in lung adenocarcinoma. The nadir criteria were developed following clinical practice guidelines, and both clinicopathologic factors and multiple characterization factors of CT were combined with ^18^F-FDG PET/CT for multivariate analysis. Our research findings indicated that there are significant differences in CT and ^18^F-FDG PET/CT features between the STAS-positive and STAS-negative groups according to univariate analysis. However, multivariate analysis revealed that the maximum tumor diameter measured by CT and the SUVmax measured by PET/CT were independent predictors of STAS. Furthermore, SUVmax showed a significant superiority over the maximum tumor diameter in predicting STAS. Our results indicate that PET/CT can serve as a non-invasive preoperative method to predict STAS in lung adenocarcinoma, and has important value in treatment decision-making.

Increasing numbers of studies now show that STAS can be used as a predictor of postoperative and healing factors in lung adenocarcinoma and was significantly linked to stronger invasive behavior [14]. In both limited and radical resection groups, STAS was identified as an independent poor prognostic factor for recurrence [6]. The routine inclusion of STAS status in pathology reports can help doctors identify high-risk patients so that they can benefit from additional interventions [15]. A substantial and robust body of scientific literature consistently demonstrates a strong correlation between the presence of STAS and reduced survival rates. Moreover, these findings consistently support the notion that STAS is an independent prognostic factor, regardless of the stage of the tumor [16]. Accurately predicting the presence of STAS in preoperative noninvasive imaging studies can aid preoperative surgical decision-making, and treatment planning and provide a basis for clinical surgery planning [17]. Previous research has indicated that CT imaging features help predict the STAS phenomenon and determine the appropriate surgical strategy before operation [18]. The current research found that the presence of STAS was statistically different in the nodule type, CTR, maximum tumor diameter, maximum solid component diameter, vasculature, pleural invasion, lobulation, and spiculation. This is consistent with Hironori's results based on a semi-quantitative assessment of early STAS in lung adenocarcinomas, which demonstrated that higher STAS was more frequent in patients with small resected solid dominant adenocarcinomas, pleural and lymphovascular invasion, and larger tumor size, and was associated with poorer recurrence-free survival [19].

The presence of STAS was identified as an independent prognostic factor in patients with radiologically pure solid lung adenocarcinoma, and the proportion of solid components was regarded as an independent imaging factor for STAS [8]. In other words, STAS was more likely to be observed in lung adenocarcinomas with a solid component compared to those without [18]. Therefore, STAS is more prevalent in predominantly solid lesions than in partially solid nodules, as well as in GGN [8]. Furthermore, a correlation exists between the type of operation and STAS. Masanori et al. discovered that patients who underwent lobectomy resection had higher levels of CTR and a higher proportion of solid tumors, as well as a higher degree of malignancy in STAS than patients who underwent sublobar resection [20]. Furthermore, STAS demonstrated a poor prognostic impact only in pure solid lesions [21]. A strong correlation exists between nodule size and STAS either. It was concluded that the STAS-positive nodules appeared to have a significantly larger size than the STAS-negative lesions [17]. According to Margerie et al., subsolid pulmonary adenocarcinomas with histological evidence of STAS were found to be larger than those without [22]. Our study found that solid tumors with a CTR of 1 commonly exhibited STAS. Multivariate prediction models indicated that maximum tumor diameter was a useful independent predictor, regardless of other CT features. Therefore, the presence of STAS suggests a higher degree of malignancy in a tumor. Taken together, Our results align with these reports, which suggest that an increase in maximum tumor diameter correlates with a higher chance of STAS occurrence, and that maximum tumor diameter can not only reflect the degree of malignancy and tumor growth but also help predict the presence of STAS. An increase in maximum tumor diameter corresponds to higher amounts of solid components, lower tumor differentiation, higher tumor activity, greater invasive ability, and more aggressive malignancy. This conclusion is consistent with the findings of Zhang, whose study of stage IA lung adenocarcinoma also confirmed that higher maximum tumor diameter and maximum solid component diameter were associated with the presence of STAS [23]. While the results showed no statistically significant difference in perineural invasion, this may reflect that STAS-positive tumor cells detach from the basement membrane of the alveolar septum at the primary site, reattach through the airway, and grow following the alveolar septum distant from the primary lesion, without relying on perineural invasion [24].

The study results indicated that all ^18^F-FDG PET/CT metrics, Namely, SUV (SUVmax, SUVmean, SUVpeak), SUL (SULmax, SULmean, SULpeak), MTV, and TLG were significantly higher in STAS-positive lesions. Additionally, half of the qualitative parameter-SUVmax showed the best predictive performance according to multivariable logistic analysis. Consistent with previous findings, a CTR ≥ 0.25 and a SUVmax ≥ 2.5 among preoperative parameters were identified as independent predictors of STAS [25]. Tumors with poor prognoses of high activity or low differentiation show increased FDG uptake due to glucose overexpression. The STAS represents anatomical tumor spread, as reflected by FDG, which represents biological aggressiveness [26]. Measuring the SUVmax of FDG uptake allows for quantitative assessment of the malignant potential of tumor cells, reflects the maximal metabolic activity of the tumor, and helps predict its biological characteristics. A higher SUVmax may be valuable in predicting the presence of STAS [25]. According to Nishimori et al., the frequency of STAS increased significantly with higher histologic grading [9]. Once STAS tumors reach a certain stage, they begin to spread through the airspace, demonstrating not only their spread, but also the increased activity of tumor cells, accelerated growth rate, and thus increased rate of ^18^F-FDG/CT uptake. Therefore, the reason for the increased SUVmax in STAS-positive lung adenocarcinomas may reflect the malignant potential and metabolic activity of their tumors. According to Nishimori, STAS is primarily linked to FDG uptake, rather than MTV or TLG, which represent the metabolically active tumor volume, and these volume parameters may not truly reflect the true state of the tumor [9]. However, some scholars that SULmax prediction is an independent prognostic factor for STAS in stage I NSCLC adenocarcinoma, possibly due to the study inclusion criteria and the fact that only SUL-related metrics were calculated and SUVmax was not considered in the study [26], and the results may need to be further confirmed.

In the multivariate logistic regression modeling study cohort, SUVmax of ^18^F-FDG PET/CT features in model 2 showed better performance than CT features of maximum tumor diameter in all aspects. Even though both maximum tumor diameter and SUVmax could indicate tumor metabolism and activity, ^18^F-FDG PET/CT is a more accurate indicator of tumor activity as the solid fraction of lung adenocarcinoma increases. Therefore, greater tumor activity and higher ^18^F-FDG uptake are associated with increased SUVmax, suggesting that lung adenocarcinoma tends to spread through the airspace. Furthermore, the presence of STAS not only increases the risk of the disease and requires more caution during clinical surgery when choosing between lobectomy, segmental resection, or wedge resection. SUVmax can be easily measured on ^18^F-FDG PET/CT and may assist in the appropriate therapeutic management of lung cancer.

This study has several limitations. Firstly, this study was a retrospective single-center study with a relatively small sample size, which may have led to selection bias and requires external validation studies. Second, this study only evaluated STAS prediction for one histologic type of lung adenocarcinoma. More histologic types of lung cancer should be included in future studies. Finally, since the present results only focus on the prediction model, further validation in clinical practice may be needed. Due to the small sample size, this preliminary study was unable to evaluate CT and PET predictors within subgroups of the same tumor stage. We recognize this as an important limitation, and will validate predictors within stage-matched groups in future research to control for potential confounding by overall stage.

## Conclusion

In conclusion, the SUVmax of ^18^F-FDG PET/CT is an imaging biomarker capable of accurately predicting STAS in lung adenocarcinoma. SUVmax showed satisfactory diagnostic performance in predicting STAS preoperatively and was superior to other measures. Predicting the presence of STAS based on preoperative information is critical for guiding treatment decisions. It also supports surgical decision-making and provides valuable information for patient survival and prognosis.

## Data Availability

The data are available from the corresponding author upon request.

## Abbreviations

^18^F-FDG: ^18^F-fluorodeoxyglucose
95%CI: 95% Confidence Interval
AUC: Area Under the Curve
CEA: Carcinoembryonic antigen
CT: Computed Tomography
CTR: Consolidation/tumor ratio
GGN: Ground Glass Nodules
LVI: Lymphovascular invasion
MIA: Minimally Invasive Adenocarcinoma
MTV: Metabolic Tumor Volume
PACS: Pictures Archived in a Communication System
PET: Positron Emission Tomography
ROC: Receiver Operating Characteristic
STAS: Spread Through Air Space
STAS ( +/-): STAS-positive/negative
SULmax/mean/peak: maximum/mean/peak Standardized Uptake value normalized by Lean body mass
SUVmax/mean/peak: maximum/mean/peak Standardized Uptake Value
TLG: Total Lesion Glycolysis
VPI: Visceral Pleural Invasion
